# The Gut–Endometriosis Axis: Genetic Mechanisms and Public Health Implications

**DOI:** 10.3390/genes16080918

**Published:** 2025-07-30

**Authors:** Efthalia Moustakli, Nektaria Zagorianakou, Stylianos Makrydimas, Emmanouil D. Oikonomou, Andreas Miltiadous, George Makrydimas

**Affiliations:** 1 Laboratory of Medical Genetics, Faculty of Medicine, School of Health Sciences, University of Ioannina, 45110 Ioannina, Greece; ef.moustakli@uoi.gr; 2 Human Computer Interaction Laboratory, Department of Informatics and Telecommunications, University of Ioannina, Kostakioi, 47150 Arta, Greece; e.oikonomou@uoi.gr (E.D.O.); a.miltiadous@uoi.gr (A.M.); 3 Department of Nursing, School of Health Sciences University of Ioannina, 4th kilometer National Highway Str. Ioannina-Athens, 45500 Ioannina, Greece; zagorianakou@uoi.gr; 4 Medical School, Aristotle University of Thessaloniki, 54124 Thessaloniki, Greece; smakrydimas@gmail.com; 5 Department of Obstetrics & Gynecology, University Hospital of Ioannina, 45500 Ioannina, Greece

**Keywords:** gut microbiota, dysbiosis, inflammation, gut–endometriosis axis, estrogen metabolism, women’s health

## Abstract

**Background/Objectives**: Endometriosis is a chronic, estrogen-driven gynecological disorder affecting approximately 10% of reproductive-aged women worldwide, with significant physical, psychosocial, and socioeconomic impacts. Recent research suggests a possible involvement of the gut microbiome in endometriosis disease mechanisms through immune manipulation, estrogen metabolism, and inflammatory networks. This narrative review aims to summarize current evidence on gut microbiota changes in endometriosis patients, explore the mechanisms by which gut dysbiosis contributes to disease progression, and examine epidemiological links between gastrointestinal health and endometriosis risk. **Methods**: A narrative review was conducted to synthesize available literature on the compositional changes in gut microbiota associated with endometriosis. The review also evaluated studies investigating potential mechanisms and epidemiological patterns connecting gut health with endometriosis development and severity. **Results**: Alterations in gut microbiota composition were observed in endometriosis patients, suggesting roles in immune dysregulation, estrogen metabolism, and inflammation. Potential gut-oriented interventions, including dietary changes, probiotics, and lifestyle modifications, emerged as promising management options. However, methodological variability and research gaps remain barriers to clinical translation. **Conclusions**: Integrating gut microbiome research into endometriosis management holds potential for improving early diagnosis, patient outcomes, and healthcare system sustainability. The study emphasizes the need for further research to address existing challenges and to develop public health strategies that incorporate microbiome-based interventions in population-level endometriosis care.

## 1. Introduction

Endometriosis is a chronic, estrogen-dependent gynecologic disease due to the implantation of endometrial-like tissue outside of the uterine cavity, most commonly involving the ovaries, fallopian tubes, and pelvic peritoneum [1]. Affecting approximately 10% of reproductive-aged women worldwide, it ranks the most common gynecologic disorders. This condition frequently causes chronic pelvic pain, dysmenorrhea, infertility, and significantly diminishes quality of life [2]. Despite its high prevalence and destructive effects, endometriosis remains underdiagnosed and understudied. Many patients experience delays in diagnosis, often ranging from 7 to 10 years after symptom onset [3,4].

In addition to having an immediate clinical impact, endometriosis is a public health concern of broader scope [5]. Comorbidities, psychosocial dimensions, and classification of chronic disease demand multidimensional approaches of care reaching far afield of conventional clinical management. The disease burden can be reduced introducing public health approaches permitting early identification, fostering increased knowledge, and delivering equitable reach of care [6]. The interest of research during the past few decades has increasingly shifted toward the human microbiota, especially toward the gut microbiome, and their roles toward disease and health. The complex community of microorganisms in the gut, known as the gut microbiota, influences hormone metabolism, systemic inflammation, and immune function [7,8]. All of these functions are intimately involved in the pathophysiology of endometriosis. Many autoimmune and chronic inflammatory disease entities have been proposed to be caused by dysbiosis, or alterations in the composition of gut microbes [9]. Immunological dysregulation, dysregulated metabolism of estrogens, and an amplified inflammatory response have been determined as a few of the mechanisms by which dysbiosis may contribute to the onset and development of endometriosis [10,11].

Elucidation of the endometriosis–gut microbiota relationship has both public health and biomedical research applications. The gut–endometriosis axis holds immense potential for developing novel disease markers for diagnosis, prevention, and the development of non-invasive treatment agents that can enhance patient outcomes [12]. Furthermore, definition of this relationship at a public health level allows incorporation of microbiome science into population-based strategies, including health education, lifestyle intervention programs, and policy development with a focus on reduction in disease burden [13].

This narrative review seeks to synthesize existing evidence regarding the association of gut microbiota with endometriosis and what public health strategies can potentially emerge from this field of research. Closing the divide between microbiome science and public health can help identify routes toward improving endometriosis prevention and management at a population level.

## 2. Methodology

### 2.1. Study Design

This narrative review synthesizes emerging evidence on the association between endometriosis and the composition on the human microbiota. While not conducted as a systematic review, a structured search approach was employed to comprehensively identify relevant literature and enhance transparency in the study selection.

### 2.2. Eligibility Criteria

Eligible studies were original, peer-reviewed research involving human participants investigating the association between endometriosis and microbiota in the gut, vagina, intestine, or reproductive tract. Only articles published in English between 1 January 2010, and 16 June 2025, were included. Exclusion criteria comprised non-original publications (e.g., reviews, meta-analyses, editorials, conference papers), studies not focused on the microbiome, research involving non-human subjects, and in vitro investigations.

### 2.3. Information Sources

The literature search was performed using two electronic databases: PubMed/Medline and Scopus. The search covered publications from 1 January 2010, to 31 December 2025, with the final search completed on 16 June 2025.

### 2.4. Search Strategy

A structured search strategy combined free-text keywords and Medical Subject Headings (MeSH) terms related to endometriosis and the human microbiome. In PubMed, the entire search query was: (“endometriosis”[MeSH Terms] OR “endometriosis”[Title/Abstract]) AND (“microbiome”[Title/Abstract] OR “microbiota”[Title/Abstract] OR “gut microbiome”[Title/Abstract] OR “intestinal microbiota”[Title/Abstract] OR “vaginal microbiome”[Title/Abstract] OR “reproductive tract microbiota”[Title/Abstract]) AND (“2010/01/01”[Date–Publication]: “2025/12/31”[Date–Publication]) AND Humans[MeSH Terms]. Although no language restrictions were applied during the search, only studies published in English were included at the screening stage.

### 2.5. Study Design

**Figure 1:**
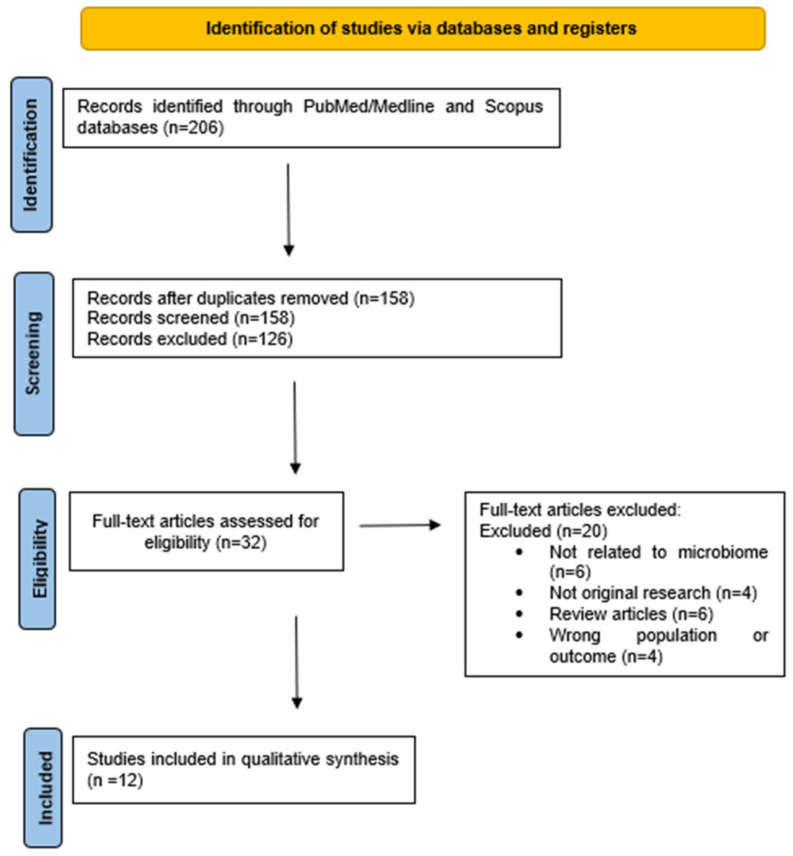
PRISMA flow diagram showing the study selection process.

After duplicate removal, 158 records were screened by title and abstract. Of these, 126 were excluded for not meeting eligibility criteria. Full-text versions of 32 articles were assessed and 20 were excluded due to lack of microbiome focus (n = 6), not being original research (n = 4), being review articles (n = 6), or involving the wrong population or outcome (n = 4). Ultimately, 12 studies met the inclusion criteria and were incorporated into this narrative review. The selection process is summarized in the flow diagram (Figure 1).

## 3. The Relationship Between Gut Microbiome and Endometriosis

### 3.1. Alterations in Gut Microbiome Composition

Endometriosis patients have demonstrated significant changes in their gut microbiome, including an imbalance in the bacterial community and a reduction in microbial diversity [14,15,16]. Characteristically, high pro-inflammatory bacterial abundance of *Escherichia coli* and *Clostridium* species has been observed. In contrast, beneficial bacteria such as *Lactobacillus* and *Bifidobacterium*, which promote gut barrier integrity and immune regulation, respectively, are decreased [17,18]. These findings are primarily derived from small-scale observational studies in human subjects, which, while valuable, limit conclusions about causality. This shift toward a dysbiotic gut environment may potentially facilitate systemic inflammation and immune dysregulation, both of which play roles in endometriosis pathogenesis. Identification of these microbial alterations sheds light on potential markers of disease as well as therapeutic targets intended to rebalance a healthy microbiome [19,20,21].

### 3.2. Mechanistic Pathways Linking Gut and Endometriosis

There is growing evidence of a potential role of gut microbiota on endometriosis development and advancement through a variety of interrelated mechanisms [22]. One important mechanism involves immunological modulation, wherein alterations in the composition of gut microbiota can disrupt immune homeostasis, potentially triggering an excessive inflammatory response that promotes the implantation and growth of ectopic endometrial tissue [20]. Dysbiosis can increase intestinal permeability, commonly referred to as ‘leaky gut’, leading to the translocation of bacterial endotoxins, such as lipopolysaccharides (LPS), into the systemic circulation. This process induces inflammation and activated immune cells, potentially facilitating the development of endometriotic lesions [23].

Additionally, the gut microbiota can affect hormonal regulation by modulating estrogen metabolism [19]. Certain gut bacteria produce enzymes such as β-glucuronidase, which deconjugate estrogens, influencing their reabsorption and circulating levels [24]. Elevated estrogen levels are a key driver of endometriotic lesion growth and persistence, directly linking microbial composition directly to disease pathophysiology [25].

These converging mechanisms, encompassing immune dysregulation, increased systemic inflammation, and altered estrogen metabolism, highlight the microbiota’s multifaceted role in endometriosis [26,27]. Unraveling these intricate host–microbiota interactions provides both a conceptual foundation and a translational pathway for the design of microbiota-targeted therapeutics as adjunctive modalities within an integrated framework for endometriosis management.

### 3.3. Evidence from Animal and Clinical Studies

Preclinical studies, primary employing murine models, have shown that modulation of the gut microbiome through probiotics and antibiotics can influence both the volume of endometriotic lesions and the associated inflammatory burden [28]. Probiotic treatment usually reduces inflammation and lesion formation, but antibiotic delivery may exacerbate these effects, indicating a critical role for microbial homeostasis in the course of disease [29,30]. While informative, these animal studies represent low-level evidence with limited generalizability to human populations.

In contrast, clinical research remains limited but suggests similar patterns. Several observational studies have reported significant differences in the gut and reproductive tract microbiota of women with endometriosis compared to healthy controls [31,32]. Early clinical trials using probiotics have reported some improvement in symptoms and inflammation, but small sample sizes and varied study designs limit definitive conclusions [33,34,35]. However, the small sample sizes, lack of blinding, and heterogeneity in study designs preclude firm conclusions at this stage.

Collectively, these findings highlight the therapeutic potential of microbiome-targeted strategies but emphasize the need for larger, rigorously designed randomized controlled trials to confirm efficacy and elucidate mechanisms.

## 4. Genetic and Epigenetic Insights into the Gut–Endometriosis Axis

### 4.1. Genetics Susceptibility Loci in Endometriosis

Genome-wide association studies (GWAS) have identified multiple genetic loci associated with an increased risk of endometriosis, highlighting the heritable nature of the disease. Notably, variants in *WNT4* (1p36.12), which is crucial for Müllerian duct development and reproductive system formation, have been strongly implicated. Polymorphisms such as rs7521902 and rs3820282 are thought to dysregulate WNT signaling, thereby facilitating ectopic endometrial implantation and lesion persistence [36,37,38]. Similarly, intronic variants within *GREB1* (2p25.1), an estrogen-responsive gene, influence chromatin organization and transcriptional activity of genes involved in cell cycle control, potentially exacerbating estrogen-driven proliferation of endometriotic tissue [39]. Other significant loci include *VEZT* (12q22), encoding a cell adhesion protein that may enhance ectopic tissue anchoring, and *FSHB* (11p14.1), involved in gonadotropin regulation and estrogen biosynthesis [40]. Together, these susceptibility loci underscore the importance of genetic variation in pathways of hormonal regulation, inflammation, and cellular adhesion in shaping individual risk for endometriosis.

Consequently, the pathophysiology of endometriosis is influenced by important pathways involving hormone control, inflammation, and cellular adhesion, which are highlighted by these genetic susceptibility loci. Crucially, new data highlights the possibility that these genetic variables may interact with the microbiota to affect immunological responses and estrogen metabolism. The intricate interaction highlighted by this gene–microbiome crosstalk will be further examined in the section that follows.

### 4.2. Gene–Microbiome Interactions and Causality

**Figure 2:**
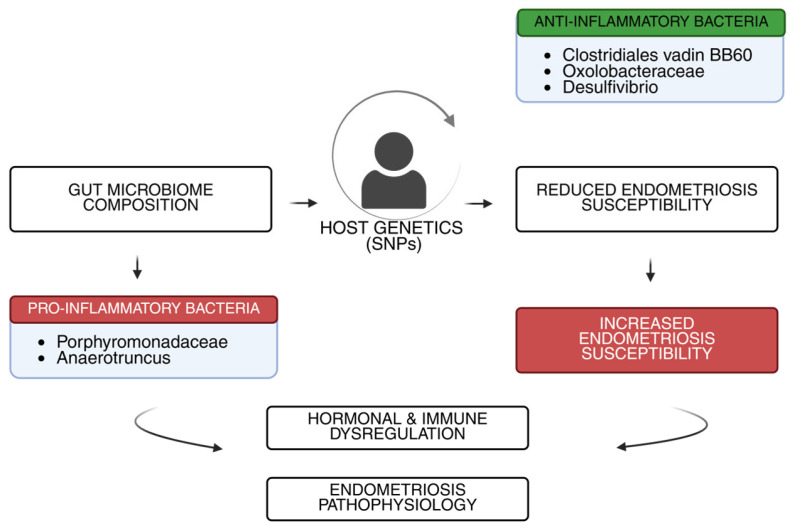
Genetic variants (SNPs) can promote either anti-inflammatory bacteria (green), which are linked to lower risk, or pro-inflammatory bacteria (red), which are linked to higher risk. This interaction contributes to immune and hormonal changes involved in endometriosis.

There is growing recognition that host genetics not only influences susceptibility to endometriosis but also shapes the composition of gut microbiota, thereby contributing indirectly to disease pathophysiology [10]. Host single-nucleotide polymorphisms (SNPs) have been shown to affect microbial diversity and abundance across populations [41]. Mendelian randomization studies suggest that genetically determined increases in anti-inflammatory bacterial groups such as *Clostridiales vadin BB60*, *Oxalobacteraceae*, and *Desulfovibrio* may lower endometriosis risk, whereas predisposition to higher levels of pro-inflammatory taxa like *Porphyromonadaceae* and *Anaerotruncus* correlates with heightened susceptibility [42]. This bidirectional relationship indicates that host genetic composition may modulate gut microbial ecosystems in ways that reinforce hormonal and immune dysregulation characteristic of endometriosis (Figure 2) [43].

### 4.3. Epigenetic and Gene Expression Regulation

Epigenetic modifications represent a critical mechanistic link between genetic predisposition, microbiome activity, and endometriosis development [44]. Aberrant DNA methylation and histone modifications have been identified in endometriotic lesions, affecting key genes involved in immune regulation, angiogenesis, and estrogen signaling [45]. Microbiota-derived metabolites, particularly short-chain fatty acids (SCFAs), are known to act as histone deacetylase inhibitors, influencing gene expression profiles in immune and endometrial cells. In parallel, dysbiosis of the estrobolome—a collection of gut microbial genes involved in estrogen metabolism—can increase β-glucuronidase activity, promoting estrogen deconjugation and reabsorption [46]. This leads to elevated systemic estrogen levels, perpetuating the growth and maintenance of ectopic endometrial tissue. These findings highlight a potential feedback loop in which host genetics and gut microbiota collaborate to sustain the inflammatory and estrogen-dependent environment of endometriosis [47].

### 4.4. Integrative Model and Future Directions

Collectively, these insights support a multifactorial model of endometriosis wherein genetic susceptibility establishes a permissive biological environment that is further influenced by gut microbiota composition and activity [20]. This gene–microbiome–epigenetic axis may amplify systemic inflammation, immune dysregulation, and hormonal imbalances, driving disease progression [48]. However, this model is primarily based on preclinical research and observational human studies, and remains largely theoretical at this stage. Future research should focus on large-scale, longitudinal studies to disentangle causal relationships between genetic variants, microbiome shifts, and endometriosis severity. Additionally, functional studies exploring gene–microbiome–metabolite interactions will be essential for identifying potential biomarkers and developing personalized microbiome-targeted therapies that take into account individual genetic backgrounds [49]. Such approaches should aim to strengthen the current evidence base through rigorous design and integration of multi-omics data.

## 5. Epidemiological Perspectives on Gut Health and Endometriosis

An increasing number of epidemiological studies are examining the association between gut health and endometriosis, while observational and population-based research has begun to clarify potential links [50] (Table 1). The prevalence or severity of endometriosis is often examined in these research in relation to differences in gut microbiota content, dietary patterns, antibiotic use, or gastrointestinal health [51]. Large population-based studies have drawn associations between inflammatory bowel disease, irritable bowel syndrome (IBS), and increased prevalence of endometriosis, suggesting common mechanisms of pathophysiology potentially mediated by gut dysbiosis [52]. As evidence of the possible involvement of an imbalance of gut microorganisms as a disease feature, observational data also point to altered bowel habits, abdominal discomfort, and other gut-origin symptoms as common in women with endometriosis [53].

Additional epidemiological studies have also explored eating habits shaping gut microbiota and their relationship with endometriosis risk [47]. Consumption of a high-fiber, fruit- and vegetable-filled eating plan, which sustains a healthy gut microbiome, has decreased risk of endometriosis, yet a high red meat and processed food eating plan can increase risk [54]. These findings support the speculation of a lifestyle-influenced gut microbiota composition having an impact on development and disease progression of endometriosis [55].

Despite these findings, there are several issues with epidemiologic studies. Confounders such as variations in diet, antibiotic use, hormone therapy, and lifestyle decisions make outcomes more difficult to interpret [56]. In addition, gut microbiota is determined by numerous variables such as genes, geography, age, and environment that may differ between comparison groups of a study. Most also rely on symptoms or diagnoses by self-reporting, a source of bias or misclassification [57].

**Table 1:** Epidemiological Insights into Gut Health and Endometriosis.

Focus Area	Key Findings	Implications
Gut Microbiota & Endometriosis [58]	Studies suggest altered gut microbiota profiles in individuals with endometriosis.	Points to a potential role of gut dysbiosis in disease development and symptomatology.
Gastrointestinal Comorbidities [59]	Associations found between endometriosis and conditions like IBS and IBD.	Suggests shared inflammatory or immune-mediated mechanisms.
Dietary Patterns [60]	High-fiber, plant-based diets linked to lower risk; red/processed meats associated with increased risk.	Diet may influence gut microbiota composition and modulate disease risk.
Antibiotic Use and Lifestyle Factors [61]	Frequent antibiotic use, hormone therapy, and lifestyle choices act as confounding variables.	These variables may obscure causal relationships in observational studies.
Study Limitations [62]	Most studies are cross-sectional and rely on self-reported data.	Limits the ability to infer causality and may introduce bias or misclassification.
Need for Future Research [63]	Longitudinal and controlled clinical studies are lacking.	Essential to determine whether gut dysbiosis is a cause or consequence of endometriosis.

Another crucial point is the cross-sectional nature of the bulk of available research, which rules out differential causal relationships between alterations of the gut microbiome and endometriosis. Longitudinal studies and tightly regulated clinical tests must cover this deficiency and shed light on whether gut dysbiosis is a precursor or a consequence of endometriosis as well as its treatment [9].

In essence, while epidemiological results increasingly suggest a connection between endometriosis and gut health, methodology limitations as well as confounders call for interpretative caution. Later research with sound designs and standardized tools of microbiome assessment will guide knowledge on gut–endometriosis interaction at a population level [32].

## 6. Public Health Implications

### 6.1. Disease Burden and Health System Impact

Endometriosis imposes a significant burden on affected individuals and healthcare systems globally [64]. Patients commonly endure debilitating symptoms such as chronic pelvic pain and infertility, which substantially impair daily functioning and quality of life. This clinical impact is further compounded by increasing healthcare utilization, including frequent visits to primary care providers, prolonged diagnostic evaluations, surgical interventions and long-term pharmacological treatment. Despite these efforts, effective symptom relief remains limited or absent in many cases [65].

A major challenge in the clinical management of endometriosis is the substantial diagnostic delay, often ranging from 7 to 10 years following symptoms onset. This delay facilitated disease progression and necessitated the use of more intensive, costly interventions. Beyond direct medical expenditures, endometriosis contributes to a considerable economic burden due to diminished workplace productivity, absenteeism, and long-term disability. Endometriosis patients frequently report recurrent absences, reduced occupational performance, and in some cases, loss of job due as a result of inadequately controlled symptoms [3,4].

Furthermore, limited accessibility to adequate diagnoses and effective therapy exacerbates health inequities and places added burdens on healthcare systems [66]. The frequent co-occurrence of endometriosis with other chronic conditions, such as irritable bowel disease and autoimmune disorders, imposes additional strain on healthcare resources [16,67]. Given the substantial disease burden and its wide-ranging impact on healthcare systems, enhancing awareness, facilitating early recognition, and implementing comprehensive, multidisciplinary models of care must be prioritized. Such areas of priority remain central to achieving optimal patient outcomes and reducing broader societal and economic costs of this complex disease [68].

### 6.2. Gut-Targeted Interventions and Preventive Strategies

Recent research on the relationship between the gut microbiota and endometriosis has put non-invasive, gut-centered public health programs in perspective as a means of preventing the disease and alleviating its symptoms [21]. Since there is proof that diet affects the composition of gut microbes and the inflammatory state of the body, changing one’s eating habits is one of the easiest and maybe most effective interventions [69]. Consumption of a high fiber, plant-heavy, and fermented foods-rich diet can promote high microbial diversity, support anti-inflammatory bacterial populations, and promote digestion health as a whole. In comparison, high red meat, ultra-processed food, and sugar consumption correlate with microbial dysbiosis and high inflammatory status, both of which can exacerbate endometriosis symptoms [70,71].

Pre- and probiotic supplementation also may offer therapeutic effects through recolonization of a healthy microbial flora, enhanced immune control, and improved intestinal barrier function [72]. While clinical proof is developing, early research has shown that select strains of *Lactobacillus* and *Bifidobacterium* may alleviate pelvic pain symptoms and reduce lesion severity [73]. Lifestyle modifications such as daily exercise, adequate sleep, and stress reduction similarly have been shown to foster gut health and reduce global inflammation, with a consequent beneficial outcome in endometriosis patients. Use of antibiotics as a form of eliminating virulent gut organisms is another area of investigation; however, this procedure must be undertaken with caution, as beneficial microbial communities may also suffer [74,75]. As we develop a clearer understanding of how the gut and endometriosis interface, incorporation of microbiome-based strategies into public health policy may facilitate more individualized, prevention-oriented, and cost-effective care alternatives within affected populations [20].

### 6.3. Translational Barriers in Microbiome Science

Significant challenges must be overcome before microbiome research may inform effective public health initiatives, even with more attention being paid to the gut microbiome’s role in endometriosis [49]. A principal challenge lies in the intrinsic complexity of host–microbiome interactions. The function and profile of gut microbiota are heavily individualized and determined by an individual’s genes, age, diet, environment, use of medication, and constellation of lifestyle choices. These variations make it difficult to develop universal biomarkers or plans of intervention with widespread efficacy across a variety of patient groups [76]. Furthermore, although promising findings have arisen from preclinical and limited clinical studies, a shortage of large, high-quality longitudinal cohorts limits interpretation of firm conclusions about causality and therapeutic efficacy [77].

There are no standardized protocols for microbiome sampling, analysis, or reporting, leading to disparities across studies and hindering meaningful comparison or synthesis of findings at larger scales [49]. In addition, rapid advancement of research on microbiomes has stayed one step ahead of development of regulatory and clinical systems of implementation [78]. While examples of probiotics and diets extensively commercialized as treating “gut health” abound, few have been rigorously shown or approved toward treating specific conditions like endometriosis. There is also a possibility of overpublicizing therapy based on microbiomes before sufficient evidence is available about efficacy and safety [31].

From a public health perspective, implementation of microbiome-based strategies will require interdisciplinarity between researchers, clinicians, policy-makers, and regulators [79]. It will also require significant expenditures on educational infrastructure involving diagnostics, surveillance, and personalizing therapy. Until effective, evidence-based guidelines become available, application of microbiome science in treating endometriosis will remain largely experimental, limiting its practical application within population-based health initiatives [80].

### 6.4. Equity, Awareness, and Access to Care

Since systemic barriers to care disproportionately affect marginalized communities, equity in the diagnosis, treatment, and public knowledge of endometriosis remains a major problem [81]. Access to specialized gynecological care, diagnostic procedures like laparoscopy, or long-term management alternatives is influenced by a person’s socioeconomic situation, race, geography, and level of health literacy [82]. According to research, women from rural areas, racial and ethnic minorities, and lower-income families frequently face longer diagnosis delays, fewer referrals to specialists, and possibly fewer treatment options because of insurance or financial limitations [83].

Moreover, both healthcare professionals and the general public often lack adequate awareness of endometriosis, contributing to the normalization of chronic pelvic pain and menstrual discomfort as routine or expected experiences. This widespread under recognition frequently results in delayed presentation and misdiagnosis, commonly as irritable bowel syndrome or psychological conditions such as anxiety [84]. Furthermore, access to diagnostic evaluations, nutritional interventions, and microbiome-informed therapeutics remains largely confined to affluent or research-intensive settings—an inequity that presents a critical barrier as scientific interest intensifies the gut–endometriosis connection [85].

A public health approach of advocacy, education, and health systems reform will reverse these inequities. Expanded provider training, health education that is culturally competent, and community outreach efforts based in communities can help reduce stigma, promote early symptom recognition, and make care paths accessible to all, including all backgrounds [86,87]. In addition, to be discussed are the broader socioeconomic determinants of health, including housing, nutrition, stress, and employment, as these have an important influence on gut health as well as chronic disease risk. Prevention of growth of existing disparities within endometriosis care includes a need to ensure new interventions, such as those of microbiomes, will be designed and delivered equitably [88].

## 7. Future Directions and Recommendations

A number of crucial priorities and possibilities for future research and public health initiatives are emerging as the intricate connection between the gut microbiota and endometriosis is further understood [89]. High-quality, multi-center, longitudinal research is desperately needed to elucidate the causal relationships between gut microbial dysbiosis and the onset, progression, and severity of endometriosis symptoms. The existing literature is largely constrained by small sample sizes and cross-sectional designs, limiting the capacity to establish casualty or temporal dynamics [28]. Advancing the field will require robust, longitudinal studies aimed at elucidating distinct microbial signatures with diagnostic or prognostic potential. The bacterial species most frequently associated with endometriosis, such as those with elevated levels of *Proteobacteria*, *Enterobacteriaceae*, *Streptococcus* spp., and *Escherichia coli*, should be included in these microbial profiles. More thorough characterization of these taxa could improve our knowledge of disease and microbial biomarkers. The development of such non-invasive biomarkers could markedly streaming diagnostic pathways and enable earlier, more individualized clinical intervention [90].

Developing targeted microbiome-based therapies is another crucial topic. Although dietary modifications, probiotics, and prebiotics have promise, randomized controlled trials are necessary to thoroughly assess their therapeutic efficacy, ideal formulations, and long-term safety [91]. Further investigation is necessary into promising treatment strategies, such as the gut microbiome’s control of β-glucuronidase activity, which is essential for estrogen deconjugation. The degree of endometriosis may be influenced by changes in the metabolic profile and circulating estrogens caused by dysbiosis-induced decreases in this enzymatic activity. Gut-focused treatments for managing and preventing endometriosis may be especially well-suited to personalized medicine approaches, which consider lifestyle variables, genetics, and individual microbiome profiles [20]. Therefore, future research should more fully examine the connection between the gut microbiota and the metabolic profile, especially estrogen levels, in endometriosis. Furthermore, converting laboratory results into practical, population-level applications will require interdisciplinary cooperation involving microbiologists, gynecologists, epidemiologists, dietitians, and public health specialists.

Integrating endometriosis and gut health into larger health promotion initiatives is crucial from a public health standpoint. In addition to stressing the importance of early detection of endometriosis symptoms, public awareness efforts should highlight the possible connection between gut health and women’s reproductive and general health [92]. Ensuring the equitable application of developing microbiome-based therapies requires policies that enable access to integrative care models, nutritional support, and affordable diagnostics [93].

To provide evidence-based, comprehensive, and prompt care, future recommendations and educational programs for healthcare professionals should take into account the growing understanding of the gut–endometriosis relationship [94]. Improving outcomes for those impacted by endometriosis will require developing research infrastructure, advocating for health equity, and closing the gap between scientific discovery and real-world application as this field expands (Table 2) [95].

## 8. Conclusions

Endometriosis constitutes a multifaceted and chronic inflammatory disorder with systemic implications that extend beyond the confines of reproductive health, posing substantial challenges to global health infrastructures. Accumulating evidence implicates the gut microbiota as a key modulator in the pathogenesis of endometriosis, operating through interrelated pathways involving immune dysregulation, dysbiosis-mediated estrogen metabolism, and persistent systemic inflammation. While current findings remain preliminary and largely derived from observational or preclinical models, they collectively underscore the potential of gut-targeted strategies—such as precision nutrition, probiotic and prebiotic innervations, and lifestyle modification—as adjunctive modalities in disease mitigation.

Nevertheless, the translational pipeline from microbiome research to clinical implementation remains underdeveloped. Addressing this gap requires large-scale, longitudinal, and mechanistically oriented studies to establish causal inferences, validate non-invasive microbial biomarkers, and define optimal therapeutic regimens with reproducible efficacy across diverse populations. Concurrently, enhanced clinical and public awareness, interdisciplinary collaboration, and equitable access to diagnostics and interventions must be prioritized to ensure widespread applicability and reduce disparities in care.

Integrating microbiome-informed approaches into endometriosis management frameworks has the potential to transform current paradigms-shifting from symptom-oriented treatment to mechanistically grounded, preventive, and personalized care. Archiving this vision will necessitate the convergence of scientific rigor, clinical innovation, and public health policy.

## Figures and Tables

**Table 2:** Key Future Directions and Recommendations.

Area	Focus	Recommendations
Research	Causality and mechanisms	Conduct longitudinal and mechanistic studies; invest in multi-omics research [32]
	Microbial biomarkers	Develop and validate non-invasive diagnostic and prognostic markers [96]
	Therapeutic interventions	Design randomized controlled trials for diet, probiotics, and prebiotics [22]
Clinical Practice	Personalized treatment approaches	Use microbiome profiling to guide individualized care [97]
	Provider education	Incorporate microbiome science into medical and allied health curricula [98]
Public Health	Awareness and education	Launch educational campaigns focused on endometriosis and gut health [99]
	Equity and access	Address structural barriers to diagnostics, dietary support, and integrative care [64]
	Policy integration	Develop guidelines and scalable models for microbiome-informed public health plans [100]

## Data Availability

Data is unavailable due to privacy or ethical restrictions.

## References

[1] Tsamantioti E.S., Mahdy H. (2025). Endometriosis. StatPearls.

[2] Cano-Herrera G., Salmun Nehmad S., Ruiz de Chávez Gascón J., Méndez Vionet A., van Tienhoven X.A., Osorio Martínez M.F., Muleiro Alvarez M., Vasco Rivero M.X., López Torres M.F., Barroso Valverde M.J. (2024). Endometriosis: A Comprehensive Analysis of the Pathophysiology, Treatment, and Nutritional Aspects, and Its Repercussions on the Quality of Life of Patients. Biomedicines.

[3] De Corte P., Klinghardt M., Von Stockum S., Heinemann K. (2025). Time to Diagnose Endometriosis: Current Status, Challenges and Regional Characteristics—A Systematic Literature Review. BJOG Int. J. Obstet. Gynaecol..

[4] Fryer J., Mason-Jones A.J., Woodward A. (2024). Understanding Diagnostic Delay for Endometriosis: A Scoping Review.

[5] Denny E., Weckesser A., Jones G., Bibila S., Daniels J., Bhattacharya S., PRE-EMPT team (2018). Women’s experiences of medical treatment for endometriosis and its impact on PRE-EMPT trial participation: A qualitative study. Pilot Feasibility Stud..

[6] Valderas J.M., Starfield B., Sibbald B., Salisbury C., Roland M. (2009). Defining Comorbidity: Implications for Understanding Health and Health Services. Ann. Fam. Med..

[7] Cardona D., Roman P. (2022). New Perspectives in Health: Gut Microbiota. Int. J. Environ. Res. Public Health.

[8] Afzaal M., Saeed F., Shah Y.A., Hussain M., Rabail R., Socol C.T., Hassoun A., Pateiro M., Lorenzo J.M., Rusu A.V. (2022). Human gut microbiota in health and disease: Unveiling the relationship. Front. Microbiol..

[9] Talwar C., Singh V., Kommagani R. (2022). The gut microbiota: A double-edged sword in endometriosis. Biol. Reprod..

[10] Guo C., Zhang C. (2024). Role of the gut microbiota in the pathogenesis of endometriosis: A review. Front. Microbiol..

[11] Nannini G., Cei F., Amedei A. (2025). Unraveling the Contribution of Estrobolome Alterations to Endometriosis Pathogenesis. Curr. Issues Mol. Biol..

[12] Baușic A.I.G., Scurtu F., Manu A., Matasariu D.R., Brătilă E. (2025). Gut Microbiota Dysbiosis in Endometriosis: A Potential Link to Inflammation and Disease Progression. Int. J. Mol. Sci..

[13] Aggarwal N., Kitano S., Puah G.R.Y., Kittelmann S., Hwang I.Y., Chang M.W. (2023). Microbiome and Human Health: Current Understanding, Engineering, and Enabling Technologies. Chem. Rev..

[14] Neri B., Russo C., Mossa M., Martire F.G., Selntigia A., Mancone R., Calabrese E., Rizzo G., Exacoustos C., Biancone L. (2023). High Frequency of Deep Infiltrating Endometriosis in Patients with Inflammatory Bowel Disease: A Nested Case-Control Study. Dig. Dis..

[15] Gan R., Yi Y., Li Y. (2023). Association of endometriosis and inflammatory bowel disease (IBD), findings from epidemiological evidence to genetic links. Fertil. Steril..

[16] Shigesi N., Kvaskoff M., Kirtley S., Feng Q., Fang H., Knight J.C., Missmer S.A., Rahmioglu N., Zondervan K.T., Becker C.M. (2019). The association between endometriosis and autoimmune diseases: A systematic review and meta-analysis. Hum. Reprod. Update.

[17] Wang Y., Yan H., Zheng Q., Sun X. (2025). The Crucial Function of Gut Microbiota on Gut–Liver Repair. hLife.

[18] Di Vincenzo F., Del Gaudio A., Petito V., Lopetuso L.R., Scaldaferri F. (2024). Gut microbiota, intestinal permeability, and systemic inflammation: A narrative review. Intern. Emerg. Med..

[19] Escorcia Mora P., Valbuena D., Diez-Juan A. (2025). The Role of the Gut Microbiota in Female Reproductive and Gynecological Health: Insights into Endometrial Signaling Pathways. Life.

[20] Datkhayeva Z., Iskakova A., Mireeva A., Seitaliyeva A., Skakova R., Kulniyazova G., Shayakhmetova A., Koshkimbayeva G., Sarmuldayeva C., Nurseitova L. (2025). The Multifactorial Pathogenesis of Endometriosis: A Narrative Review Integrating Hormonal, Immune, and Microbiome Aspects. Medicina.

[21] Wang M., Liu W., Zheng L., Ma S., Jin L., Zhao D., Li D. (2025). Broadening horizons: Microbiota as a novel biomarker and potential treatment for endometriosis. Front. Microbiol..

[22] Chadchan S.B., Naik S.K., Popli P., Talwar C., Putluri S., Ambati C.R., Lint M.A., Kau A.L., Stallings C.L., Kommagani R. (2023). Gut microbiota and microbiota-derived metabolites promotes endometriosis. Cell Death Discov..

[23] Rondanelli M., Borromeo S., Cavioni A., Gasparri C., Gattone I., Genovese E., Lazzarotti A., Minonne L., Moroni A., Patelli Z. (2025). Therapeutic Strategies to Modulate Gut Microbial Health: Approaches for Chronic Metabolic Disorder Management. Metabolites.

[24] Hu S., Ding Q., Zhang W., Kang M., Ma J., Zhao L. (2023). Gut microbial beta-glucuronidase: A vital regulator in female estrogen metabolism. Gut Microbes.

[25] Baker J.M., Al-Nakkash L., Herbst-Kralovetz M.M. (2017). Estrogen–gut microbiome axis: Physiological and clinical implications. Maturitas.

[26] Wang Z., Zhang L., Liu X., Xu L. (2025). The role of reproductive tract microbiota in gynecological health and diseases. J. Reprod. Immunol..

[27] Pérez-Prieto I., Rodríguez-Santisteban A., Altmäe S. (2024). Beyond the reproductive tract: Gut microbiome and its influence on gynecological health. Curr. Opin. Clin. Nutr. Metab. Care.

[28] Xholli A., Cremonini F., Perugi I., Londero A.P., Cagnacci A. (2023). Gut Microbiota and Endometriosis: Exploring the Relationship and Therapeutic Implications. Pharmaceuticals.

[29] Yang S., Qiao J., Zhang M., Kwok L.Y., Matijašić B.B., Zhang H., Zhang W. (2025). Prevention and treatment of antibiotics-associated adverse effects through the use of probiotics: A review. J. Adv. Res..

[30] Petrariu O.A., Barbu I.C., Niculescu A.G., Constantin M., Grigore G.A., Cristian R.E., Mihaescu G., Vrancianu C.O. (2024). Role of probiotics in managing various human diseases, from oral pathology to cancer and gastrointestinal diseases. Front. Microbiol..

[31] Hearn-Yeates F., Horne A.W., O’Mahony S.M., Saunders P.T.K. (2024). Microbiome: The impact of the microbiota–gut–brain axis on endometriosis-associated symptoms: Mechanisms and opportunities for personalised management strategies. Reprod. Fertil..

[32] Li Y., Li Y., Ouyang D., Liu L., Ren D., Wu X. (2025). Association between endometriosis and gut microbiota: Systematic review and meta-analysis. Front. Microbiol..

[33] Cruz Mosquera F.E., Perlaza C.L., Naranjo Rojas A., Murillo Rios S., Carrero Gallego A., Fischersworring S.I., Rodríguez J.S., Liscano Y. (2025). Effectiveness of Probiotics, Prebiotics, and Symbiotic Supplementation in Cystic Fibrosis Patients: A Systematic Review and Meta-Analysis of Clinical Trials. Medicina.

[34] Nista E.C., Parello S., Brigida M., Amadei G., Saviano A., De Lucia S.S., Petruzziello C., Migneco A., Ojetti V. (2025). Exploring the Role of Gut Microbiota and Probiotics in Acute Pancreatitis: A Comprehensive Review. Int. J. Mol. Sci..

[35] Yassine F., Najm A., Bilen M. (2025). The role of probiotics, prebiotics, and synbiotics in the treatment of inflammatory bowel diseases: An overview of recent clinical trials. Front. Syst. Biol..

[36] Luong H.T., Painter J.N., Shakhbazov K., Chapman B., Henders A.K., Powell J.E., Nyholt D.R., Montgomery G.W. (2013). Fine mapping of variants associated with endometriosis in the WNT4 region on chromosome 1p36. Int. J. Mol. Epidemiol. Genet..

[37] Nyholt D.R., Low S.K., Anderson C.A., Painter J.N., Uno S., Morris A.P., MacGregor S., Gordon S.D., Henders A.K., Martin N.G. (2012). Genome-wide association meta-analysis identifies new endometriosis risk loci. Nat. Genet..

[38] Matalliotakis M., Zervou M.I., Matalliotaki C., Rahmioglu N., Koumantakis G., Kalogiannidis I., Prapas I., Zondervan K., Spandidos D.A., Matalliotakis I. (2017). The role of gene polymorphisms in endometriosis. Mol. Med. Rep..

[39] Chadchan S.B., Popli P., Liao Z., Andreas E., Dias M., Wang T., Gunderson S.J., Jimenez P.T., Lanza D.G., Lanz R.B. (2024). A GREB1-steroid receptor feedforward mechanism governs differential GREB1 action in endometrial function and endometriosis. Nat. Commun..

[40] Angioni S., D’Alterio M.N., Coiana A., Anni F., Gessa S., Deiana D. (2020). Genetic Characterization of Endometriosis Patients: Review of the Literature and a Prospective Cohort Study on a Mediterranean Population. Int. J. Mol. Sci..

[41] Blekhman R., Goodrich J.K., Huang K., Sun Q., Bukowski R., Bell J.T., Spector T.D., Keinan A., Ley R.E., Gevers D. (2015). Host genetic variation impacts microbiome composition across human body sites. Genome Biol..

[42] Liu Z., Chen P., Luo L., Liu Q., Shi H., Yang X. (2023). Causal effects of gut microbiome on endometriosis: A two-sample mendelian randomization study. BMC Women’s Health.

[43] Uzuner C., Mak J., El-Assaad F., Condous G. (2023). The bidirectional relationship between endometriosis and microbiome. Front. Endocrinol..

[44] Marquardt R.M., Tran D.N., Lessey B.A., Rahman M.S., Jeong J.W. (2023). Epigenetic Dysregulation in Endometriosis: Implications for Pathophysiology and Therapeutics. Endocr. Rev..

[45] Kobayashi H., Imanaka S., Yoshimoto C., Matsubara S., Shigetomi H. (2024). Rethinking the pathogenesis of endometriosis: Complex interactions of genomic, epigenetic, and environmental factors. J. Obstet. Gynaecol. Res..

[46] Mirzaei R., Kavyani B., Nabizadeh E., Kadkhoda H., Asghari Ozma M., Abdi M. (2023). Microbiota metabolites in the female reproductive system: Focused on the short-chain fatty acids. Heliyon.

[47] Qin R., Tian G., Liu J., Cao L. (2022). The gut microbiota and endometriosis: From pathogenesis to diagnosis and treatment. Front. Cell Infect Microbiol..

[48] Bacaloni S., Agrawal D.K. (2025). Nutrition, Gut Microbiota, and Epigenetics in the Modulation of Immune Response and Metabolic Health. Cardiol. Cardiovasc. Med..

[49] Yang S.Y., Han S.M., Lee J.Y., Kim K.S., Lee J.E., Lee D.W. (2025). Advancing Gut Microbiome Research: The Shift from Metagenomics to Multi-Omics and Future Perspectives. J. Microbiol. Biotechnol..

[50] Dang C., Chen Z., Chai Y., Liu P., Yu X., Liu Y., Liu J. (2024). Assessing the relationship between gut microbiota and endometriosis: A bidirectional two-sample mendelian randomization analysis. BMC Women’s Health.

[51] Martire F.G., Costantini E., d’Abate C., Capria G., Piccione E., Andreoli A. (2025). Endometriosis and Nutrition: Therapeutic Perspectives. J. Clin. Med..

[52] Nabi M.Y., Nauhria S., Reel M., Londono S., Vasireddi A., Elmiry M., Ramdass P.V. (2022). Endometriosis and irritable bowel syndrome: A systematic review and meta-analyses. Front. Med..

[53] Salmeri N., Sinagra E., Dolci C., Buzzaccarini G., Sozzi G., Sutera M., Candiani M., Ungaro F., Massimino L., Danese S. (2023). Microbiota in Irritable Bowel Syndrome and Endometriosis: Birds of a Feather Flock Together-A Review. Microorganisms.

[54] Szczepanik J., Dłużewska M. (2024). The Importance of Diet in the Treatment of Endometriosis. Women.

[55] Svensson A., Brunkwall L., Roth B., Orho-Melander M., Ohlsson B. (2021). Associations Between Endometriosis and Gut Microbiota. Reprod. Sci..

[56] Baryakova T.H., Pogostin B.H., Langer R., McHugh K.J. (2023). Overcoming barriers to patient adherence: The case for developing innovative drug delivery systems. Nat. Rev. Drug Discov..

[57] Pedroza Matute S., Iyavoo S. (2023). Exploring the gut microbiota: Lifestyle choices, disease associations, and personal genomics. Front. Nutr..

[58] Yang H. (2024). The causality between gut microbiota and endometriosis: A bidirectional Mendelian randomization study. Front. Med..

[59] Zondervan K.T., Becker C.M., Koga K., Missmer S.A., Taylor R.N., Viganò P. (2018). Endometriosis. Nat. Rev. Dis. Primer.

[60] Parazzini F., Viganò P., Candiani M., Fedele L. (2013). Diet and endometriosis risk: A literature review. Reprod. Biomed. Online.

[61] Parasar P., Ozcan P., Terry K.L. (2017). Endometriosis: Epidemiology, Diagnosis and Clinical Management. Curr. Obstet. Gynecol. Rep..

[62] Leonardi M., Hicks C., El-Assaad F., El-Omar E., Condous G. (2020). Endometriosis and the microbiome: A systematic review. BJOG Int. J. Obstet. Gynaecol..

[63] Ata B., Yildiz S., Turkgeldi E., Brocal V.P., Dinleyici E.C., Moya A., Urman B. (2019). The Endobiota Study: Comparison of Vaginal, Cervical and Gut Microbiota Between Women with Stage 3/4 Endometriosis and Healthy Controls. Sci. Rep..

[64] Ellis K., Munro D., Clarke J. (2022). Endometriosis Is Undervalued: A Call to Action. Front. Glob. Women’s Health.

[65] Carbone M.G., Campo G., Papaleo E., Marazziti D., Maremmani I. (2021). The Importance of a Multi-Disciplinary Approach to the Endometriotic Patients: The Relationship between Endometriosis and Psychic Vulnerability. J. Clin. Med..

[66] Jindal M., Chaiyachati K.H., Fung V., Manson S.M., Mortensen K. (2023). Eliminating health care inequities through strengthening access to care. Health Serv. Res..

[67] Shafrir A.L., Palmor M.C., Fourquet J., DiVasta A.D., Farland L.V., Vitonis A.F., Harris H.R., Laufer M.R., Cramer D.W., Terry K.L. (2021). Co-occurrence of immune-mediated conditions and endometriosis among adolescents and adult women. Am. J. Reprod. Immunol..

[68] Pónusz-Kovács D., Pónusz R., Sántics-Kajos L.F., Csákvári T., Kovács B., Várnagy Á., Kovács K.A., Bódis J., Boncz I. (2024). Evaluation of the Epidemiological Disease Burden and Nationwide Cost of Endometriosis in Hungary. Healthcare.

[69] Aziz T., Hussain N., Hameed Z., Lin L. (2024). Elucidating the role of diet in maintaining gut health to reduce the risk of obesity, cardiovascular and other age-related inflammatory diseases: Recent challenges and future recommendations. Gut Microbes.

[70] Rinninella E., Tohumcu E., Raoul P., Fiorani M., Cintoni M., Mele M.C., Cammarota G., Gasbarrini A., Ianiro G. (2023). The role of diet in shaping human gut microbiota. Best Pract. Res. Clin. Gastroenterol..

[71] Tomova A., Bukovsky I., Rembert E., Yonas W., Alwarith J., Barnard N.D., Kahleova H. (2019). The Effects of Vegetarian and Vegan Diets on Gut Microbiota. Front. Nutr..

[72] Chandrasekaran P., Weiskirchen S., Weiskirchen R. (2024). Effects of Probiotics on Gut Microbiota: An Overview. Int. J. Mol. Sci..

[73] Latif A., Shehzad A., Niazi S., Zahid A., Ashraf W., Iqbal M.W., Rehman A., Riaz T., Aadil R.M., Khan I.M. (2023). Probiotics: Mechanism of action, health benefits and their application in food industries. Front. Microbiol..

[74] Lalla A.T., Onyebuchi C., Jorgensen E., Clark N. (2024). Impact of lifestyle and dietary modifications for endometriosis development and symptom management. Curr. Opin. Obstet. Gynecol..

[75] Varghese S., Rao S., Khattak A., Zamir F., Chaari A. (2024). Physical Exercise and the Gut Microbiome: A Bidirectional Relationship Influencing Health and Performance. Nutrients.

[76] Abavisani M., Khoshrou A., Foroushan S.K., Ebadpour N., Sahebkar A. (2024). Deciphering the gut microbiome: The revolution of artificial intelligence in microbiota analysis and intervention. Curr. Res. Biotechnol..

[77] Mansfield L., Ramponi V., Gupta K., Stevenson T., Mathew A.B., Barinda A.J., Herbstein F., Morsli S. (2024). Emerging insights in senescence: Pathways from preclinical models to therapeutic innovations. Npj Aging.

[78] Juarez V.M., Montalbine A.N., Singh A. (2022). Microbiome as an immune regulator in health, disease, and therapeutics. Adv. Drug Deliv. Rev..

[79] Kamel M., Aleya S., Alsubih M., Aleya L. (2024). Microbiome Dynamics: A Paradigm Shift in Combatting Infectious Diseases. J. Pers. Med..

[80] Davenport S., Smith D., Green D.J. (2023). Barriers to a Timely Diagnosis of Endometriosis: A Qualitative Systematic Review. Obstet. Gynecol..

[81] Giudice L.C., Oskotsky T.T., Falako S., Opoku-Anane J., Sirota M. (2023). Endometriosis in the era of precision medicine and impact on sexual and reproductive health across the lifespan and in diverse populations. FASEB J..

[82] Levy H., Janke A. (2016). Health Literacy and Access to Care. J. Health Commun..

[83] Hoagland A., Kipping S. (2024). Challenges in Promoting Health Equity and Reducing Disparities in Access Across New and Established Technologies. Can. J. Cardiol..

[84] Kocas H.D., Rubin L.R., Lobel M. (2023). Stigma and mental health in endometriosis. Eur. J. Obstet. Gynecol. Reprod. Biol. X.

[85] Ser H.L., Au Yong S.J., Shafiee M.N., Mokhtar N.M., Ali R.A.R. (2023). Current Updates on the Role of Microbiome in Endometriosis: A Narrative Review. Microorganisms.

[86] Tulchinsky T.H., Varavikova E.A. (2014). Expanding the Concept of Public Health. The New Public Health.

[87] Blenner S.R., Lang C.M., Prelip M.L. (2017). Shifting the Culture Around Public Health Advocacy: Training Future Public Health Professionals to Be Effective Agents of Change. Health Promot. Pract..

[88] Cockerham W.C., Hamby B.W., Oates G.R. (2017). The Social Determinants of Chronic Disease. Am. J. Prev. Med..

[89] Griffiths M.J., Horne A.W., Gibson D.A., Roberts N., Saunders P.T.K. (2024). Endometriosis: Recent advances that could accelerate diagnosis and improve care. Trends Mol. Med..

[90] Kimber-Trojnar Ż., Pilszyk A., Niebrzydowska M., Pilszyk Z., Ruszała M., Leszczyńska-Gorzelak B. (2021). The Potential of Non-Invasive Biomarkers for Early Diagnosis of Asymptomatic Patients with Endometriosis. J. Clin. Med..

[91] Roy S., Dhaneshwar S. (2023). Role of prebiotics, probiotics, and synbiotics in management of inflammatory bowel disease: Current perspectives. World J. Gastroenterol..

[92] Garmendia J.V., De Sanctis C.V., Hajdúch M., De Sanctis J.B. (2025). Endometriosis: An Immunologist’s Perspective. Int. J. Mol. Sci..

[93] Gulliver E.L., Young R.B., Chonwerawong M., D’Adamo G.L., Thomason T., Widdop J.T., Rutten E.L., Rossetto Marcelino V., Bryant R.V., Costello S.P. (2022). The future of microbiome-based therapeutics. Aliment. Pharmacol. Ther..

[94] Rogers P.A.W., Adamson G.D., Al-Jefout M., Becker C.M., D’Hooghe T.M., Dunselman G.A.J., Fazleabas A., Giudice L.C., Horne A.W., Hull M.L. (2017). Research Priorities for Endometriosis. Reprod. Sci..

[95] Rahmioglu N., Zondervan K.T. (2024). Endometriosis: Disease mechanisms and health disparities. Bull. World Health Organ..

[96] Huang L., Liu B., Liu Z., Feng W., Liu M., Wang Y., Peng D., Fu X., Zhu H., Cui Z. (2021). Gut Microbiota Exceeds Cervical Microbiota for Early Diagnosis of Endometriosis. Front. Cell. Infect. Microbiol..

[97] Pai A.H.Y., Wang Y.W., Lu P.C., Wu H.M., Xu J.L., Huang H.Y. (2023). Gut Microbiome–Estrobolome Profile in Reproductive-Age Women with Endometriosis. Int. J. Mol. Sci..

[98] Roullier C., Sanguin S., Parent C., Lombart M., Sergent F., Foulon A. (2021). General practitioners and endometriosis: Level of knowledge and the impact of training. J. Gynecol. Obstet. Hum. Reprod..

[99] Le Busque B., Mellish S. (2023). Endometriosis Awareness Month on Social Media: A Content Analysis of Images and Captions on Instagram. Women.

[100] Li Z., Yin Z., Chen W., Wang Z. (2025). Impact of Gut and Reproductive Tract Microbiota on Estrogen Metabolism in Endometriosis. Am. J. Reprod. Immunol..

